# Examinations for Soundness1Read before the February meeting of the New York County Veterinary Medical Association.

**Published:** 1899-03

**Authors:** J. E. Ryder

**Affiliations:** New York City


### EXAMINATIONS FOR SOUNDNESS.[1]

BY J. E. RYDER, D.V.S.,
NEW YORK CITY.

IT is not my intention to-night to enter into the general details of examining horses for soundness, but to present to you, briefly, three points of unsoundness upon which we frequently disagree, which I cannot say is to our credit, considering our knowledge of these conditions, for this difference in opinion is not likely to increase the confidence of the general public in our profession.

Before taking up either of my points of unsoundness, I wish to say that my interpretation of the term "sound horse" is an animal

[1] Read before the February meeting of the New York County Veterinary Medical Association.

without acute or chronic pathological condition or lesion; "practical soundness," an animal with a chronic pathological lesion, but which is not likely to interfere with his usefulness. Under these definitions of soundness the conditions I am going to present to you will be unsound.

*Splints.* Adhering to the term sound, an animal with a splint must be condemned; but the question so often in dispute is, "When can an animal with a splint be passed practically sound?" That we take into consideration several factors in reaching a decision of this kind is admitted by all. At the same time the public should know that certain factors do exist which may either condemn or pass the animal practically sound. I will admit that a large number of horses are working every day and going sound with splints, at the same time we know a great many do not.

Dealers, almost without exception, claim to their customers that a splint is not an unsoundness, and our profession has made no effort to enlighten the purchaser upon the subject; in fact we have placed him in a position at the present time more bewildering than ever. We have done this by one veterinarian passing the horse without mentioning the splint, or, perhaps, calling it a blemish, while another condemns him outright or passes him practically sound.

For practical soundness in examining an animal with a splint, the following factors should be taken into consideration, viz.: First, age of the animal; second, size of the splint; third, its location; fourth, class or breed of animal (work required); fifth, action of the animal; sixth, gait of the animal.

An animal under six years of age should always receive a very close examination, all factors being carefully considered, and the animal not to receive the benefit of doubt.

The location of a splint is of the utmost importance in determining his serviceability. If situated close to or at the knee he should be condemned; if on the posterior border of the rudimentary metacarpal, condemned, as should also the commonly called pegged splint, that form which extends across the posterior surface of the principal as well as the rudimentary metacarpal bones. The exceptionally low splint should also receive close attention on account of its great tendency to interfere with the action of the flexor tendons. The knee splint is condemned on account of its tendency to involve that articulation, and that on the posterior border of the rudimentary metacarpal and the pegged splint on account of interference with the action of the flexor tendons. Some practitioners claim that the tendons will adapt themselves to their changed position in a short time and the animal go sound, but my experience has been otherwise.

Size also has important bearing upon practical soundness; an exceptionally large splint should always be regarded with suspicion; its location is immaterial, and unless all other factors are exceptionally favorable, should condemn.

In harness- and saddle-horses the purchaser should receive the benefit of all doubts, as the work required of this class is of the kind likely to cause a recurrence of splint-lameness, while in the draught-horse, who is never or seldom asked to go off a walk, the practitioner can be more lenient.

Action is of great importance, and the following should be taken into consideration, viz.: Does he go high, and does he strike the ground hard? The higher his action and the harder he strikes the ground the greater will be the tendency for him to reproduce periostitis, lameness, and increased development of the splint.

Does he wind, paddle, travel close, toe in, etc.? In examining a horse with a splint, notice all the irregularities in his gait—some I have mentioned, and many others I have not; if any irregularities exist, notice if likely to cause him to interfere or hit himself; if so, condemn. I know that a great many horses with an irregularity of gait will go sound for an indefinite period; but you do not know at what moment the horse will interfere and reproduce periostitis, lameness, and increased development.

I also realize that a great many horses with splints in a bad location and faulty action give good service. On the other hand, many do not, and the chances are against them; therefore, I believe we are justified in condemning on the grounds I have mentioned, and if we do not our clients are not receiving justice at our hands. As for a splint being classed as a blemish, I do not believe that any pathological lesion which may under certain conditions cause lameness can be put down as a blemish.

*Spavin.* When does a coarse hock become a spavin?

Not many years ago the term "coarse hock" was comparatively unknown—that is, outside of our profession, but of recent years it has become alarmingly common, and one used to cover a multitude of sins. Dealers will claim, and I regret to say some practitioners will pass, a horse with the term "coarse hock," when he has a spavin that can be photographed a block away, providing he goes anywhere near sound at the time.

A coarse hock is one with well-developed cuneiform bones, possibly with the same well-developed condition of the principal and rudimentary metatarsal bones; in other words, they are more rough and prominent around this articulation than usual. I may add that at the present time a coarse hock of this kind in a horse six years old or older is more rare than a true spavin.

In examining for soundness it is necessary that we make a correct differential diagnosis between a coarse sound hock and one spavined and unsound, in justice to both buyer and seller, for we will all admit that a true coarse hock will stand as much wear and tear as a smooth one, and frequently more. We should also bear in mind that it is common to notice in three- and four-year olds a pronounced coarseness of this articulation, and that with proper care they will fine down by the time they reach the sixth year.

Granting what I have said, I do not believe that a finely bred horse with smooth, even articulations at other points can have a pronounced normal coarseness of one or both hocks, neither do I believe that a horse can have a pronounced enlargement or coarseness, if you like, of one hock, and a fine, smooth hock on the opposite side.

The number of spavined horses sold at the present time as being coarse in one or both hocks cannot be imagined by one not in close touch with the sale marts. Again, it is almost beyond one's comprehension how some men can and will stand up and propound theories regarding this condition in order to justify themselves in passing these animals. One case comes to my mind, and is as follows, viz.: Several years ago a stallion of international reputation was condemned for spavin by two or three of our most able practitioners, while two or three others passed him sound with a coarse hock, one of whom advanced the theory that animals of this breed, especially the entire horses, were always exercised on a lunge rein which threw their weight from the centre and almost entirely upon one side, and on account of this a coarseness developed to compensate the articulation for the increased weight it was called upon to carry, forgetting in his argument that animals exercised in this manner are reversed every few minutes, which causes the weight to be continually shifting from side to side. Again, if this condition did develop in one or both legs it would be nothing more or less than an exostosis (spavin), due to the periostitis which the weight caused, if it caused anything.

You will also notice that some practitioners condemn horses when this coarseness is situated on or near the anterior surface of the hock, and pass a more pronounced condition of the same kind if situated on the internal surface near the posterior border. I believe that this is wrong; if a coarseness is sufficient to be called a spavin at one point, it is at another; because it is not so conspicuous does not change its pathology or its tendency to cause future trouble.

In examining a horse whose hocks are suspicious, and where you are in doubt as to the true condition, examine him first cold, drive him, and again when cooled out, trotting him to the halter on each examination; also watch him closely when backing from the stall, and turn him short both sides. You may also flex his hocks strongly for a few minutes and then trot him, and if you fail to detect any soreness or irregularity of action at any time during these examinations or manœuvres, both hocks showing you an equal amount of coarseness or very nearly so and then not pronounced, I believe you have a coarse-hocked and sound horse.

*Curb.* When is a horse with a curb practically sound?

First, all animals, the condition of the curb immaterial, under six years of age should be condemned.

Second, an animal of any age with round-bone and a curby conformation (cow hocks) should be condemned.

Third all large curbs, and especially those soft and accompanied with a thickening of the surrounding tissues, should be condemned.

An animal over six years of age, all conditions being equal, has the advantage over a young and immature one.

An animal with a good, straight leg and well-developed hock, over six years of age, having a curb of long standing which is small and hard, and who shows no lameness or irregularity of action either when cold, driving, warm, or after cooling out, can be passed practically sound.

A condition frequently met with, and one often causing a difference of opinion, is where the rudimentary metatarsal and cuboid bones extend around upon the posterior border of the leg beyond their usual distance. This condition should be examined very carefully, and if no thickening or enlargement of the sheath of the tendon or exostosis on the bones can be detected, he should be passed as sound; but if this condition exists in an animal of curby conformation, even if no true curb exists at the time, I believe he should be condemned, for we know that if an animal of this kind receives the least hard work a curb will be developed, which will be very unsatisfactory to the purchaser and no credit to the examiner.

I have omitted the pathology of these conditions, as we all agree upon it, and have attempted to bring before you in as few words as possible the conditions as they exist and as we meet them when examining for soundness, and I hope that some rule or understanding can be reached by which we can agree, and not render opinions directly opposite to each other as so often happens at the present.

